# “If I knew I could get that every hour instead of alcohol, I would take the cannabis”: need and feasibility of cannabis substitution implementation in Canadian managed alcohol programs

**DOI:** 10.1186/s12954-021-00512-5

**Published:** 2021-06-23

**Authors:** Bernie Pauly, Meaghan Brown, Clifton Chow, Ashley Wettlaufer, East Side Illicit Drinkers Group for Education (EIDGE), Brittany Graham, Karen Urbanoski, Russell Callaghan, Cindy Rose, Michelle Jordan, Tim Stockwell, Gerald Thomas, Christy Sutherland

**Affiliations:** 1 Canadian Institute for Substance Use Research, University of Victoria, Technology Enterprise Facility, 2300 McKenzie Ave, Victoria, BC V8P 5C2 Canada; 2 School of Nursing, University of Victoria, Victoria, BC Canada; 3 Vancouver Coastal Health, Victoria, BC Canada; 4 Centre for Addiction and Mental Health, Toronto, Ontario Canada; 5 Vancouver Area Network of Drug Users (VANDU), Vancouver, BC Canada; 6 British Columbia Centre for Disease Control, Vancouver, BC Canada; 7 School of Public Health and Social Policy, University of Victoria, Victoria, BC Canada; 8 Northern Medical Program, University of Northern British Columbia (UNBC), Prince George, BC Canada; 9 School of Population and Public Health, University of British Columbia (UBC), Vancouver, BC Canada; 10 Canadian Mental Health Association Sudbury/Manitoulin, Sudbury, Ontario Canada; 11 Shelter House, Thunder Bay, Ontario Canada; 12 Department of Psychology, University of Victoria, Victoria, BC Canada; 13 Ministry of Health, Province of British Columbia, Victoria, BC USA; 14 PHS Community Services Society, Vancouver, BC Canada; 15 Faculty of Medicine, University of British Columbia, Vancouver, BC Canada

**Keywords:** Managed alcohol programs, Alcohol harm reduction, Harm reduction, Cannabis substitution, Homelessness, Severe alcohol use disorder

## Abstract

**Background:**

While there is robust evidence for strategies to reduce harms of illicit drug use, less attention has been paid to alcohol harm reduction for people experiencing severe alcohol use disorder (AUD), homelessness, and street-based illicit drinking. Managed Alcohol Programs (MAPs) provide safer and regulated sources of alcohol and other supports within a harm reduction framework. To reduce the impacts of heavy long-term alcohol use among MAP participants, cannabis substitution has been identified as a potential therapeutic tool.

**Methods:**

To determine the feasibility of cannabis substitution, we conducted a pre-implementation mixed-methods study utilizing structured surveys and open-ended interviews. Data were collected from MAP organizational leaders (*n* = 7), program participants (*n* = 19), staff and managers (*n* = 17) across 6 MAPs in Canada. We used the Consolidated Framework for Implementation Research (CFIR) to inform and organize our analysis.

**Results:**

Five themes describing feasibility of CSP implementation in MAPs were identified. The first theme describes the characteristics of potential CSP participants. Among MAP participants, 63% (*n* = 12) were already substituting cannabis for alcohol, most often on a weekly basis (*n* = 8, 42.1%), for alcohol cravings (*n* = 15, 78.9%,) and withdrawal (*n* = 10, 52.6%). Most MAP participants expressed willingness to participate in a CSP (*n* = 16, 84.2%). The second theme describes the characteristics of a feasible and preferred CSP model according to participants and staff. Participants preferred staff administration of dry, smoked cannabis, followed by edibles and capsules with replacement of some doses of alcohol through a partial substitution model. Themes three and four highlight organizational and contextual factors related to feasibility of implementing CSPs. MAP participants requested peer, social, and counselling supports. Staff requested education resources and enhanced clinical staffing. Critically, program staff and leaders identified that sustainable funding and inexpensive, legal, and reliable sourcing of cannabis are needed to support CSP implementation.

**Conclusion:**

Cannabis substitution was considered feasible by all three groups and in some MAPs residents are already using cannabis. Partial substitution of cannabis for doses of alcohol was preferred. All three groups identified a need for additional supports for implementation including peer support, staff education, and counselling. Sourcing and funding cannabis were identified as primary challenges to successful CSP implementation in MAPs.

## Background

### Alcohol-related harms and MAPs

Internationally, 3 million deaths (5.3% of all deaths) and 132.6 million disability-adjusted life years (DALYs) can be attributed to alcohol annually [1]. In spite of its legal status, alcohol is associated with a range of social and physical harms to individuals and the public. “Acute” physical harms from alcohol include poisonings and unintentional injuries, while the list of “chronic” physical harms and alcohol-attributable diseases is lengthy, including liver disease, cancers, strokes, and gastrointestinal diseases [2]. These harms generally increase with a dose–response relationship to volume of alcohol consumption [2]. “Social” harms include issues with housing, financial, relationships, legal, and workplace difficulties. Social and physical harms combined result in substantial costs to the Canadian social and health systems, exceeding the costs of tobacco and other substances such as opioids, sedatives, and cocaine [3]. “Risky” drinking occurs across the population with social acceptance of drinking practices informally regulated by social and cultural norms. This is apparent when comparing social acceptance toward binge drinking among privileged populations and in leisure settings (e.g., public binge drinking among college and university students) [4] to stigma and discrimination toward visible binge drinking among people experiencing poverty and/or homelessness [5]. Indeed, the costs and distribution of these harms are shaped by socioeconomic, political, and other contextual factors, although may be more publicly visible and concentrated among those with high levels of structural disadvantage [6–9].

While alcohol use disorders (AUDs) are experienced by 3–4% of the global population, rates of up to 37.9% have been found among males experiencing homelessness [1, 10]. People who consume alcohol daily in the context of homelessness cite both positive and negative impacts of alcohol, for example in staving off daily risks of withdrawal, contributing to community connection with other people who drink, recreation, and to address psychological pain and trauma [11, 12]. Heavy alcohol use can also be understood as one way in which people experiencing homelessness cope and survive within the structurally violent conditions that contribute to and exacerbate homelessness [13, 14]. Within abstinence-based health and housing systems, people experiencing homelessness paired with AUD describe cycles of displacement and discrimination within and between services, insecurity of personal safety and belongings, and loss of connection to social supports such as friends, family and for some Indigenous people, ancestral communities [5, 15]. “Street-based illicit drinking” refers to the consumption of alcohol in harmful or otherwise stigmatized ways, including the use of non-beverage alcohol (e.g., hand sanitizer, mouthwash), often in situations where safer forms of regulated alcohol are unaffordable or unavailable [5, 16]. Harms associated with street-based illicit drinking extend beyond consumption itself to include harms associated with consumption in criminalized spaces, including policing and charges related to public intoxication, targeted criminalization related to racism, and violence [17].

Managed Alcohol Programs (MAPs) have emerged as a therapeutic option to reduce harms associated with co-occurring homelessness and severe AUD in Canada and internationally [18–20]. There are over 22 programs currently operating in Canada. MAPs vary in their programming but typically offer some or all of the following: (1) an alcohol management intervention where safer, more regulated forms of alcohol are provided to address patterns of binge and street-based drinking; (2) provision of shelter, temporary, or permanent housing; (3) integrated health care; and (4) social and cultural programming [18]. In Indigenous-led or informed programs, a MAP is often integrated within a broader program grounded in Indigenous worldviews and knowledge.

The Canadian Managed Alcohol Program Study (CMAPS) is a national longitudinal mixed-methods controlled study evaluating 8 MAPs across Canada. Findings from this research have demonstrated benefits including reductions in some alcohol-related harms (e.g., withdrawal seizures, legal, housing issues), reduced use of non-beverage alcohol, improved quality of life and safety, increased housing stability, and reduced health and criminal justice system costs. In a cross-sectional analysis comparing 6 MAP sites, Stockwell et al. [21] present findings on the drinking patterns and alcohol-related harms among MAP participants (*n* = 175) and matched local controls (*n* = 189). Admission to a MAP was associated with benefits, with new MAP participants (< 2 months in the program) experiencing fewer acute physical harms (e.g., seizures, passing out, health issues) and social harms (e.g., contact with police, relationship issues) relative to controls. MAP participants had a lower total volume of alcohol per drinking day and reported fewer days of drinking non-beverage alcohol compared to controls. Analyses of qualitative data from CMAPS illustrate that MAPs are spaces for healing and recovery for participants, who are frequently displaced and criminalized on the street and within largely abstinence-based housing and health services [19]. Participants describe MAPs as safe environments that reduce precarity associated with homelessness and severe AUD in abstinence-based systems, enhance social integration and connection to community and family, provide a sense of purpose and shared ownership, and for some Indigenous participants, promote connection to Indigenous identity and culture (when cultural supports are provided) [15, 22, 23].

Despite the documented benefits of MAPs, there are concerns related to chronic elevated risk of alcohol-related diseases with sustained, heavy consumption of alcohol without periods of abstinence. Findings from a longitudinal study by Stockwell et al. [24] indicate similar reductions in alcohol use between MAP participants and controls over 6–12 months (mean drinks per day: MAP = − 8.11, *p* < 0.001; controls = − 8.54, *p* < 0.001; mean drinking days per month: MAP = − 2.51 days, *p* < 0.05; control = − 4.81 days, *p* = 0.0001). These findings indicate that MAP participants consume alcohol in a more even, less sporadic, and binge pattern than controls, with total alcohol consumption spread out over more days (25.41 vs 19.64 days per month, *p* < 0.001). Longitudinal analyses of liver function during periods on and off MAP also demonstrate deterioration in liver function among participants who leave MAP. However, there is evidence that regular breaks from metabolizing alcohol can be protective against liver disease and some liver specialists recommend all drinkers should have abstinent days each week [25]. With these findings in mind, MAP participants may benefit from access to adjunctive alcohol harm reduction therapies, alongside alcohol management, to reduce the potential for harmful unintended consequences of continuous alcohol administration.

### Cannabis substitution for alcohol

While cannabis use is not without harm, the scale of harms is substantially lower than for alcohol [26, 27]. The most well-studied cannabis-related harms include potential for cognitive developmental impacts, particularly for youth and for people with a pre-disposition for psychotic disorders [28, 29], and risk of motor vehicle collisions [30]. Cannabis consumption via smoke inhalation is associated with similar respiratory inflammation and injury processes as tobacco, and there is a need for additional controlled clinical studies to clarify pulmonary harms [31]. Despite these concerns, at this time the profile of harms associated with cannabis appears to be less pronounced than that of alcohol.

Observational and retrospective studies on patterns of cannabis use among people who use illicit substances, alcohol and/or tobacco have contributed to a growing body of evidence of the potential therapeutic benefits of cannabis [32–38]. Cross-sectional surveys of medical cannabis users have shown reductions in self-reported use of alcohol, opioids, tobacco, prescription drugs, and other illicit substances [33, 35]. Therapeutic use of cannabis has been found to be subjectively beneficial for pain, anxiety, and sleep [28]. Cannabis use may offer additional benefits for people with severe AUD who commonly experience chronic pain, insomnia, and appetite loss, all of which are frequently identified as reasons for use among medical cannabis users [35, 39]. Cannabis also has the potential to reduce alcohol cravings and withdrawal symptoms [40–42] and may play a role in mitigating the inflammatory impacts of alcohol [43] as well as hepatic harms [44].

Currently, Cannabis Substitution Programs (CSPs) are not formally offered in MAPs. In a review of research on cannabis substitution for alcohol, Subbaraman [45] concluded that cannabis met or partially met six out of seven criteria identified by Chick and Nutt [46] for substitution therapy. They concluded that evidence for cannabis substitution therapy should be considered premature due to a lack of longitudinal and controlled trials that assess risk among people with AUD. In order to prepare for a pilot study and potential longitudinal trial, we undertook a feasibility study of CSPs in MAPs.

## Purpose and objectives

This feasibility study is situated within CMAPS, a mixed-methods program of research evaluating the effectiveness, implementation, and impacts of MAPs in multiple Canadian cities. Initiated in 2011 in collaboration with community non-profit agencies operating MAPs, people with lived experience, regional and provincial health planners, CMAPS aims to generate evidence to support the development and implementation of practices and policies for MAPs in different settings. In 2019, we initiated discussions with MAP organizational leaders on the potential for CSPs within their programs. Representatives from 7 sites expressed interest in exploring the feasibility of formal implementation and evaluation of CSPs in MAPs.

The objectives of this feasibility study were to (1) examine sample characteristics, suitability, and acceptability of cannabis substitution in MAPs from the perspectives of participants, staff, managers and organizational leaders, (2) explore practical, legal, financial and ethical issues, and (3) develop a rigorous protocol for conducting a pilot and controlled trial study. In this paper, we specifically address objective #1 and present the perspectives of MAP participants, staff, managers and organizational leaders on the sample characteristics, suitability, and acceptability of cannabis substitution in MAPs. We will explore practical, legal, financial and ethical issues elsewhere and are developing a rigorous protocol for future conduct of pilot and controlled trial study.

## Methods

Applying principles of Community-Based Participatory Research (CBPR), we built upon existing relationships with MAP organizational leaders, staff, and managers and people with lived expertise to conceptualize and implement this study and assist with interpretation of findings. To scope out the potential for a study of CSPs in MAPs, we engaged the CMAPS Community of Practice (CoP), a knowledge mobilization platform which includes 350+ members (including MAP providers, decision makers, and persons with lived expertise) across Canada. Organizational leaders at 7 MAPs and the East Side Illicit Drinkers Group for Education (EIDGE), an independent group of people with lived expertise affiliated with VANDU (Vancouver Areas Network of Drug Users) committed to partnering on this research. We used the Consolidated Framework for Implementation Research (CFIR) to develop data collection tools and inform the analysis. Interview guides and preliminary findings were presented to MAP organizational leaders and EIDGE members, and their feedback was incorporated as the study progressed.

### Theoretical framework

The CFIR has been applied to research in a broad range of settings, including substance use and public health interventions. It consists of 39 theoretical constructs grouped into five domains: the characteristics of individuals, the intervention, process of implementation, inner and outer context. While CFIR provides a framework for formative evaluation of intervention implementation, it can also be applied to guide feasibility assessments pre-implementation to optimize intervention effectiveness [47]. In a systematic review, Kirk et al. [48] found only 2 (of 26) studies using the CFIR focused on pre-implementation and concluded that its limited use for this purpose was a missed opportunity. The CFIR can help to investigate implementation issues before an intervention is operationalized and can be used to inform program design, identify potential barriers, refine the implementation strategy, and adapt the intervention prior to implementation [48]. We aimed to systematically identify and explore constructs relevant to CSP implementation, thereby increasing the likelihood of successful implementation.

We present findings on MAP participant, staff, and leadership perspectives on the feasibility of CSPs in MAPs, grouped into the four of the five CFIR domains, including: (1) characteristics of program participants, (knowledge and beliefs about the intervention, individual stage of change, and other personal attributes such as current alcohol and cannabis use patterns); (2) characteristics of the CSP (perspectives of participants, staff, and managers on cannabis substitution models including dosing, type of cannabis, and route of administration); (3) inner setting (infrastructure, culture, available resources, and access to knowledge and information); and (4) outer setting (funding and sustainability). For the purposes of this pre-implementation study, we did not investigate constructs relevant to the domain “processes of implementation” as this domain  is more appropriate to post-implementation contexts and evaluation purposes. This approach is consistent with Damschroder et al.’s [47] recommendations to tailor the application of specific constructs to the scope and context of the study.

### Sample and recruitment

We recruited 7 organizational leaders across 7 MAPs and then proceeded with recruitment of participants, staff, and managers at 6 MAPs where organizational leaders provided support in principle for piloting a CSP. We recruited MAP participants using purposive sampling based on the recommendations from staff and managers, who identified participants likely to engage in a potential CSP (*n* = 19, including 3–4 participants per program). The majority of participants identified as male (*n* = 15, 78.9%) and Indigenous (*n* = 11, 57.9%), with a minority of participants identifying as white (*n* = 8, 42.1%). Mean age was 46 years old (range 34–57 years). Participants were typically long-term residents of MAPs with a mean length of 4.4 years. All participants met the criteria for probable AUD using the Alcohol Use Disorders Inventory Test (AUDIT; mean score = 27.6, range 14–35). MAP participants received an honouraria of $25 in cash for the interview. We also interviewed 17 staff members from the 6 programs, including frontline housing and shelter workers, case managers, supervisors, and program managers.

MAP staff, management, and organizational leaders were recruited via email invitations sent by the research assistant. Participation was voluntary. Written informed consent was obtained from all participants. Ethical approval for this study was obtained from the University of Victoria Research Ethics Board (REB; #13-002).

### Data collection and analysis

Interviews were conducted to gather MAP participant, staff and organizational leadership perspectives on the acceptability, practicality, and suitability of cannabis substitution and approaches to evaluation in MAPs. Separate interview guides were developed for the three participant groups. Interview guides were structured according to the five CFIR domains (noted above). Closed and open-ended questions were developed to reflect CFIR constructs salient to the pre-implementation context and MAP setting in each of the four domains identified above.

Interview guides with MAP participants were designed with input from EIDGE to ensure appropriate framing and questions. A MAP organizational leader and collaborator on the study team reviewed staff and organizational leader interview guides. Interviews with participants lasted approximately one hour and were audio recorded. Interviewers received training from the research coordinator (CC) or PI (BP).

Interviews with MAP participants were conducted by a research associate (AW) and included questions about current alcohol and cannabis use, substitution practices and factors affecting substitution, perceived need and impacts of cannabis use, and preferred dosing, timing, and modes of administration. Participants were asked to rank their preference and willingness to participate in proposed intervention options including either complete cannabis substitution, where cannabis completely substitutes alcohol, or partial substitution, where some of the alcohol they currently receive from the program is substituted with cannabis. For the partial substitution model, three sub-models were also ranked: (1) administration of cannabis, partially replacing alcohol, at set times by staff, (2) administration of cannabis at set times by staff with a participant choosing either alcohol or cannabis, and (3) daily dispensing of cannabis with self-administration throughout the day. For the complete substitution model, participants ranked sub-models (1) and (3). Open-ended questions focused on the relative advantages and disadvantages of these options and recommendations for adapting and integrating CSPs into MAPs.

The research coordinator (CC) conducted interviews with MAP organizational leaders, managers, and staff. Interviews with organizational leaders focused on knowledge of cannabis substitution for alcohol, willingness to support cannabis substitution, and relevant barriers and facilitators to implementation in the inner and outer settings. Interviews with staff and managers included questions regarding knowledge, attitudes and beliefs about cannabis, self-efficacy to offer treatment, current practice related to cannabis, and processes such as mode and timing of administration. Staff were also asked to rank the partial and complete substitution models and sub-models, and to identify perceived advantages and disadvantages of the proposed intervention options and factors relevant to the inner and outer settings.

Data summaries were created for each interview question and grouped according to the  four CFIR domains. Descriptive statistics including mean frequencies were generated for closed-ended questions, including current volume of alcohol and cannabis use, current substitution practices, and preference and willingness to participate in the proposed cannabis substitution models. Descriptive statistics (mean frequencies) were also generated for common responses to open ended questions to identify key factors relevant to implementation from the perspective of participants and staff. We analyzed responses to the open-ended questions using content analysis to develop data summaries, which highlighted facilitators and barriers to implementation and differences in perspectives among the three participant groups. Data were also stratified by program site to account for important contextual factors in interpretation. Findings are aggregated to protect participant and program confidentiality.

## Results

We present findings according to key themes reflecting four domains of the CFIR. Each theme identifies determinants influencing implementation at the organizational or collective level: (1) If I have cannabis, I will use cannabis (characteristics of CSP participants); (2) Prioritizing choice and tailoring programs (CSP intervention); (3) Existing Constraints, Essential Supports, and Meeting people where they are at (inner setting)); and (4) Lack of core funding and sustainable cannabis supply (outer setting).

### If I have cannabis, I will use cannabis (individual characteristics)

This theme includes findings pertaining to CFIR constructs: personal attributes (participant-identified needs for cannabis substitution, current patterns and rationales of use); knowledge and beliefs about CSP (perceived benefits and harms of cannabis and cannabis substitution), and individual stage of change (willingness to participate in cannabis substitution and individual needs and program goals). In this theme, we specifically focused on potential CSP participants as they are already part of the MAP program and the success of the program is contingent on their willingness and interest in the intervention. Further, we prioritized MAP participant perspectives over staff consistent with principles of harm reduction rooted in social justice [49] and our overall approach in collaborating with people with lived and living experience.

#### Current cannabis use

Current cannabis use was commonly reported among MAP participants with 53% (*n* = 10) reporting daily use and 32% (*n* = 6) weekly use. Three participants (16%) reported not currently using cannabis. Smoking cannabis without tobacco via joint was the most popular method (68%, *n* = 13). Only a small minority of MAP participants used cannabis in non-combustible forms, specifically as edibles (11%, *n* = 2).

Overall 63.2% (*n* = 12) of MAP participants reported using cannabis as a substitution method, although at varying frequencies. About a third of participants reported never having used cannabis for alcohol substitution (35%, *n* = 7). MAP participants most often used cannabis as an alcohol substitution strategy on a weekly basis (42%, *n* = 8). Access to cannabis or alcohol influenced participants’ abilities to practice cannabis substitution, either by choice or by necessity in order to address alcohol cravings and/or withdrawal:

"When I don't have any money for alcohol. A couple times a month I guess […] If I have cannabis, I'll use cannabis. If I have alcohol, I'll use alcohol." (R3303)

In the above quote, the participant reported never using cannabis to substitute for alcohol when alcohol is available. Other MAP participants described using cannabis to reduce alcohol consumption when cannabis is offered to them by friends or available by other means. This participant reported daily cannabis substitution in an effort to reduce alcohol use:

“Daily, Okay. Yeah, because like I said, I can go to bed for a couple hours and miss drinks and not care. Right. And then somebody say “want to smoke a joint?” and I say “all right, go for that! […] You know, and I don't miss it. I don't miss a drink. I'm trying to wean off the alcohol. I really am.” (R6303)

Most participants indicated using cannabis for alcohol cravings (78.9%, *n* = 15; Table 1), such as when securing money for alcohol is difficult or to “take the edge off” (R5302) in waiting for the next alcohol dose. Most also indicated using cannabis for alcohol withdrawal symptoms (52.6%, *n* = 10). Several participants reported using cannabis early in the morning before the first pour or voiced a desire to use cannabis in the morning. These reports highlight existing individual alcohol harm reduction practices through cannabis substitution among MAP participants, despite a lack of formalized CSPs.

**Table 1:** Current cannabis use patterns and substitution practices

MAP participants	*n* = 19 (%)
*Frequency of use*	
Daily or almost daily	10 (52.6%)
Weekly	6 (31.6%)
Monthly	0
Less thank monthly	0
Never	3 (15.8%)
*Preferred method of use*	
Smoking, no tobacco	13 (68.4%)
Smoking, with tobacco	2 (10.5%)
Edible	2 (10.5%)
Vaping	0
Tincture or oil	0
NA/not using cannabis	2 (10.5%)
*Frequency of cannabis substitution*	
Daily or almost daily	2 (10.5%)
Weekly	8 (42.1%)
Monthly	1 (5.3%)
Less than monthly	1 (5.3%)
Never	7 (36.8%)
*Cannabis use for alcohol withdrawal*^a^	
Yes	15 (78.9%)
No	4 (21.1%)
*Cannabis use for alcohol cravings*^a^	
Yes	10 (52.6%)
No	8 (42.1%)
NA	1 (5.3%)

^a^MAP participants were asked if they ever use cannabis for alcohol withdrawal or cravings

#### Willingness and perceived need for cannabis substitution

Interviews with program participants highlighted a strong willingness to participate in a formalized CSP. Among MAP participants, 84% (*n* = 16) stated they would participate in some type of CSP offered in MAPs (Table 3). Among MAP staff, 88.2% (*n* = 15) identified CSPs as a specific need in MAPs.

MAP participants were asked an open-ended question about their personal goals related to drinking in order to assess alignment of a CSP with self-identified needs (Table 2A). Participant goals were primarily to stop drinking (36.8%), reduce drinking (21.1%), or drink safer (10.5%). Four of 19 MAP participants (21.1%) identified no goals related to their drinking. Other goals in MAP included employment and/or recreation (5.3%, *n* = 1).

**Table 2:** A: Self-identified MAP participant goals related to drinking and B: self-identified estimated impact of cannabis substitution on alcohol consumption

MAP participants	*n* = 19 (%)
*A. Self-identified goals*	
Stop drinking	7 (36.8%)
Reduce drinking	4 (21.1%)
Safer drinking	2 (10.5%)
Other (e.g., work, recreation)	1 (5.3%)
None	4 (21.1%)
NA	1 (5.3%)
*B. Estimated impact of cannabis substitution on alcohol consumption*	
Decrease consumption	11 (57.9%)
Increase consumption	2 (10.5%)
No change to consumption	4 (21.1%)
Don’t know	1 (5.3%)
NA	1 (5.3%)

The majority of participants felt that cannabis would help them to reduce drinking (57.9%, *n* = 11) (Table 2B), although some cited availability and costs of cannabis as barriers to their current capacity to access cannabis at an ideal amount for substitution:

“It would decrease it. Oh God yeah. If I knew I could get that every hour instead of alcohol, I would take the cannabis." (R6302)

A minority felt that a CSP would have no substitution effect. While 21.1% (*n* = 4) felt their drinking would remain the same, only 10.5% (*n* = 2) felt that their drinking would increase with concurrent cannabis use.

#### Perceived benefits and harms of cannabis substitution

Beyond impacts to drinking patterns, MAP participants and staff identified other potential benefits of cannabis substitution in MAPs. Among MAP participants, 36.8% highlighted cannabis use as a strategy for increasing appetite often impacted by chronic alcohol use. Nutrition and increases in appetite have been commonly cited as impacts of MAPs in previous research [15], although participants in this study identified ongoing concerns with their appetite and food intake:

“Our eating habits. You know, get hungry. A lot of people don't eat because they’re drinking all the time…” (R6303)

MAP participants also appreciated the ‘mellow’ effect of some strains of cannabis and compared the mood altering effects they experience and witness in others with alcohol versus cannabis. They remarked that cannabis use seemed to reduce conflict and potential violence among themselves and their peers:

"You seem to be able to catch a buzz and not get drunk. You don't get all discombobulated. You start swinging (getting into fights) with alcohol but not with pot." (R5301)

"I really don't see people getting violent when they're smoking marijuana. It seems to be more a social thing. I don't see people fighting. Doesn't seem to cause aggression. It seems to mellow people out. Alcohol…I've never met a happy drunk. There's always aggression. Some people that are cooked on a doobie, the worst thing is they're going to empty your fridge." (R5302)

Others commented on the social benefits of cannabis including enhanced functionality, the ability to remember conversations, communicate clearly, work or participate in recreation:

“I'm still like, coherent you know, I'm not like when you drink like you’re falling down drunk, like I used to be, you know. And when I smoke weed, I'm still able to have whole conversations and remember things. Alcohol removes all that shit. It does. Cannabis is far better for this." (R6302)

Other reasons for and benefits of cannabis use among MAP participants included perceived improvements in mental health, such as anxiety, stress, ADHD, and depression (31.6%, *n* = 6) and sleep (31.6%, *n* = 6). While more participants associated cannabis with benefits to mental and social health, 15.8% (*n* = 3) of MAP participants felt that cannabis may have negative impacts on the mental health of themselves or others, such as worsening psychosis and cognitive impacts (e.g., difficulty with focus and attention). One participant identified over sedation and co-intoxication with alcohol as a potential concern. Two participants felt that cannabis provided in a dried form may impact respiratory health.

Overall, MAP participants viewed cannabis as an acceptable and feasible alternative to alcohol with some benefits over alcohol. Interestingly, the information they reported aligns with current benefits and harm of cannabis. One potential issue would be if cannabis is additive to alcohol rather than being used as a substitution.

### Prioritizing choice and tailoring supports (intervention characteristics)

This theme includes findings pertaining to the program participant, staff, and manager perspectives on models for cannabis substitution (i.e., partial or complete substitution), including details on cannabis administration and associated benefits and harms.

#### Cannabis substitution models: preferences and willingness to substitute

When asked about their willingness to partially or completely substitute cannabis for alcohol, most MAP participants were willing to participate in a partial substitution model (78.9%, *n* = 15), while 63.2% (*n* = 12) of participants would be willing to participate in complete substitution (Table 3). Ratings for willingness and preference for complete substitution were relatively lower than partial substitution, although several participants entertained the possibility of transitioning completely to cannabis in the future. As one participant stated: “No. No substitution. For now anyway. It may change in a year or two.” (R5302). Participants who were interested in completely substituting cannabis for alcohol most often expressed preference for slow titration from partial to complete substitution at their own pace and comfort.

**Table 3:** Cannabis substitution models: participant and staff perceptions

MAP participants	*N* = 19 (%)	Residential/shelter MAPs *N* = 16 (%)
*Willingness to participate*		
CSP, no model specified		
Yes	16 (84.2%)	
No	2 (10.5%)	
Don’t know	1 (5.3%)	
CSP, partial substitution		
Yes	15 (78.9%)	
No	3 (16%)	
Don’t know	1 (5.3%)	
CSP, complete substitution		
Yes	12 (63.2%)	
No	5 (26.3%)	
Don’t know	2 (10.5%)	
*Willingness to participate in CSP-Sub-models*		
Partial substitution, staff administration at fixed times	15 (78.9%)	15 (93.8%)
Partial substitution, staff administration by participant choice	13 (68.4%)	13 (81.3%)
Partial substitution, self-administration	11 (57.9%)	8 (50.0%)
Complete substitution, staff administration at fixed times	9 (47.4%)	9 (56.3%)
Complete substitution, self-administration	6 (31.6%)	4 (25.0%)
NA		
*Preference of CSP sub-models(1st ranked option)*		
Partial substitution, staff administration at fixed times	3 (15.8%)	3 (18.8%)
Partial substitution, staff administration by participant choice	5 (26.3%)	5 (31.3%)
Partial substitution, self-administration	6 (31.6%)	3 (18.8%)
Complete substitution, staff administration at fixed times	0	0
Complete substitution, self-administration	4 (21.1%)	4 (25.0%)
NA	1 (5.3%)	1 (6.3%)

MAP staff	N = 17 (%)	*N* = 14 (%)
*Feasibility of CSP sub-models (1st ranked option)*		
Partial substitution, staff administration at fixed times	5 (29.4%)	5 (35.7%)
Partial substitution, staff administration by participant choice	7 (41.2%)	7 (50.0%)
Partial substitution, self-administration	3 (17.6%)	0
Complete substitution, staff administration at fixed times	0	0
Complete substitution, self-administration	0	0

Comparing the partial substitution sub-models, MAP participants were most *willing* to participate via staff administration of cannabis at fixed dosing times (78.9%, *n* = 15), followed by staff administration with a choice to receive alcohol or cannabis (68.4%, *n* = 13), and self-administration (57.9%, *n* = 12). Alternatively, they indicated they most *preferred* self-administration (31.2%, *n* = 6), with only 3 participants (15.8%) preferring staff administration of cannabis at fixed dosing times. Participants who preferred the option of self-administration spoke to the importance of autonomy and choice. Others who preferred staff administration of cannabis noted that staff were a necessary support to prevent binge use and co-intoxication in the program:

"I don't know if that would work very good. People’d be sharing it and splitting it and selling it? Yeah. It wouldn't work out. It'd be like giving us three bottles of wine in the morning and saying here you go for the rest of the day." (R6304)

“I haven't seen anyone succeed with that. I've seen people get their set amount [of alcohol] and you know, they're jonesing half way through the day cause they used it all up […] For marijuana for some people it might work, but I don't think that will work for everybody.” (R5303)

As the above quotes illustrate, MAP participants who were skeptical about a self-administration model voiced concerns about rationing cannabis use throughout the day for themselves and/or others. The option for staff administration, with a choice of alcohol or cannabis as needed and at specific administration times, was more often seen as a compromise between participant-driven dosing and staff support for safer or more moderate use:

“That might be a little more like suitable for my kinda...Well, because if I wanted the drink then I could choose to have it. I shouldn't been told what I have to do.” (R4302)

"That would be perfect. Well, it’s hard to say because the wine’s uh…I more prefer wine than anything, definitely people want better choices and I think this would be a good choice for everybody […] (self-administration of cannabis) wouldn't work. Because it'd be gone at 830 in the morning. No, I mean, a joint an hour or something like that would be sufficient." (R6303)

Variation in programmatic contexts accounted for differences in preference and willingness across CSP sub-models. Drop-in programs, where daily allotments of alcohol are given to clients at the start of the day and self-administered, ruled out the possibility of models where staff administer cannabis throughout the day to clients. As such, participants in drop-in programs were not asked to consider staff administration models. In the sub-sample of participants in residential and shelter-based MAPs where staff administer alcohol (*n* = 16), the most preferred option was partial substitution via staff administration with participant choice of receiving alcohol or cannabis (31.3%, *n* = 5), with 18.8% (*n* = 3) of participants preferring partial substitution via self-administration. This highlights differences in feasibility of CSP sub-models across programs, with the most feasible options in terms of preference and willingness being staff administration models in residential and shelter-based programs and self-administration models in drop-in programs.

MAP staff unanimously felt that a partial substitution model would be most feasible. Staff also ranked the partial substitution model with staff administration and participant choice as the most feasible option for participant engagement (41.1%, *n* = 7). This model was seen to be a ‘client-centered’ approach that would allow for participants to substitute alcohol for cannabis at their own pace, while also allowing staff to assist participants as they transition into cannabis substitution.

MAP staff did not rule out the possibility of complete substitution as a feasible model, although they often questioned the capacity of their programs to support complete substitution, particularly for drop-in or day MAPs that cannot provide around the clock support. Several recommended a progression model that allows participants to move from partial to complete substitution depending on choice.

#### Cannabis type and route of administration: there are some deal breakers

Cannabis types and routes of administration were ranked in terms of preference (MAP participants) and feasibility (MAP staff; Table 4). Most MAP participants showed a preference for dry cannabis via joint or smoking (73.7%, *n* = 14). Some perceived that smoking cannabis is associated with a ‘better buzz,’ in addition to benefits such as rapid onset and immediate pain management. The familiarity of dried cannabis and social aspects of smoking were also identified as key benefits.

**Table 4:** Cannabis types and routes of administration: preference and feasibility

MAP participants	*N* = 19 (%)
*Cannabis type and route preference (1st ranked)*	
Dry cannabis, smoked	14 (73.7%)
Dry cannabis, vaporized	0
Cannabis oil, vaporized	0
Cannabis with nicotine, vaporized	0
Cannabis capsules, oral	3 (15.8%)
Cannabis edibles, oral	1 (5.3%)
Choice of multiple options per day	0
NA	1 (5.3%)
*Cannabis type and route preference (frequency of rank within top 3 options)*^a^	
Dry cannabis, smoked	14 (73.7%)
Dry cannabis, vaporized	4 (21.1%)
Cannabis oil, vaporized	6 (31.6%)
Cannabis with nicotine, vaporized	2 (10.5%)
Cannabis capsules, oral	9 (47.4%)
Cannabis edibles, oral	11 (61.1%)
Choice of multiple options per day	2 (10.5%)
NA	1 (5.3%)
*Cannabis type and route “deal breakers”*^a^	
Dry cannabis, smoked	2 (10.5%)
Dry cannabis, vaporized	4 (21.1%)
Cannabis oil, vaporized	3 (15.8%)
Cannabis with nicotine, vaporized	8 (42.1%)
Vaping in general	4 (21.1%)
Cannabis capsules, oral	4 (21.1%)
Cannabis edibles, oral	1 (5.3%)
Other	1 (5.3%)
No deal breaker	5 (26.3%)
NA	1 (5.3%)

MAP staff	*N* = 17 (%)
*Feasibility of cannabis type and route (1st ranked option)*	
Dry cannabis, smoked	6 (35.3%)
Dry cannabis, vaporized	0
Cannabis oil, vaporized	2 (11.8%)
Cannabis with nicotine, vaporized	0
Cannabis capsules, oral	5 (29.4%)
Cannabis edibles, oral	3 (17.6%)
Choice of multiple options per day	1 (5.9%)
NA	0

^a^Participants could rank up to seven options

Staff often estimated that dried cannabis would be the most feasible option because of a sense that participants preferred this form and would consequently be more likely to engage. Staff also highly ranked capsules and edibles as feasible options, identifying that by-laws requiring outdoor smoking of cannabis could increase risks associated with sharing individually tailored doses and products. Staff and managers also perceived capsules and edibles as safer options that would allow staff to witness administration.

While many MAP participants identified oral forms of cannabis such as edibles (61.1%, *n* = 11) and capsules (47.4%, *n* = 9) within their top three preferences, a minority of participants identified these options as ‘deal breakers’ for participation in cannabis substitution (Table 4). However, some participants illustrated their preferences for oral forms of cannabis as respiratory harm reduction alternatives. For a minority of participants, vaping as a smoking alternative was identified as a ‘deal breaker’ for their participation in the CSP (*n* = 4, 21.1%), although a larger minority ranked vaping of cannabis oil within their top three options (*n* = 6, 31.6%). Contextually at the time of data collection, widespread and misleading media coverage of rare vaping-related lung disease [50] due to illegal additives may have negatively influenced participant perceptions of vaping.

### Inner setting

In this section, we report on CFIR sub-constructs relevant to the inner setting of MAPs including existing infrastructure (storage, space, and human resource capacity); available resources (human resource and social support needs); access to knowledge and information (cannabis training and education needs); and culture (organizational support for CSP and harm reduction).

#### Existing constraints and essential supports

MAP participants, staff and managers, and organizational leaders identified many material and non-material factors relevant to implementation. Most staff and organizational leaders felt that there was adequate space for safe storage of cannabis and relevant equipment. By-laws prohibiting smoking indoors raised concerns among staff about spaces for cannabis consumption during the winter. Staff and managers repeatedly identified requirements for human resources (i.e., ‘staffing’), education, and training as essential to CSP implementation.

#### Human resource and social support needs

MAP participants were asked to identify factors that may facilitate their success in a CSP. Most commonly, MAP participants requested enhanced social supports parallel to CSP implementation. The following participant spoke to the value of engaging with peers who are also part of the CSP:

"Probably another member who was using the edible beside me. Making sure, obviously, taking care. Making sure everything's going okay.” (R1303).

Like the participant in this quote, several participants identified social supports for coping with changes associated with transitioning from alcohol to cannabis including peer support groups, connection with family, and counselling. MAP participants also felt that they would benefit from regular health assessments if they were to take part in a CSP.

MAP staff and managers spoke to significant human resource constraints and a need for staff workload relief. Management often highlighted restrictions to staff capacity and that additional responsibilities, such as cannabis preparation, administration, and clinical support, would be beyond current capacity:

"I think we need more staffing right now. I think having…I think we could do it but to have more staff would obviously be more consistent and actually…um…not only with cannabis dispensing but with alcohol dispensing and just with med dispensing to have another staff here would be...would make a huge difference and maybe a lot of things would be more consistent. So yes, we could do it, I just...like I said it gets so busy in here, I don’t know sometimes we're just running around and like forget to write something down...” (S1201)

“I mean, if we are not funded by the [health authority] moving forward for medical staff that might be something else that we have to look at. But I mean, I'm just hoping that that's going to happen. I'm just assuming that's going to happen." (S2201)

As these staff describe, MAPs often operate with precarious funding and potentially with limitations in staffing support. Several staff spoke to existing needs for enhanced clinical staffing, such as to address a lack of daily nursing support in some MAPs. Additionally, staff requested clinician support with knowledge on cannabis therapeutics and monitoring, particularly in programs where clinical support is currently limited.

#### Education and training needs: “Smart Serve for pot”

Almost all MAP staff and management requested education and training resources on monitoring and safety of cannabis administration, “like Smart Serve [alcohol administration training] for pot” (S3204). Suggested training topics included assessment of intoxication and other cannabis-related harms (including protocols and assessment tools), cannabis botany, strains and dosing, operation of cannabis equipment such as vaporizers, and appropriate cannabis administration practice and documentation. Several staff commented on existing complexities in assessing intoxication when program participants use cannabis and are requesting an alcohol pour, as one staff described::

"Overall intoxication - sometimes some of our residents, we don’t serve them when they're under the influence, but some of them seem to think that they're only under the influence, if they're drinking more alcohol than they were provided. They don't realize that the assessment done is based on intoxication, whether that be from alcohol or marijuana or the combination of both."—S6202

Staff also requested education and access to evidence on cannabis substitution for both staff and participants, including indications, contraindications, benefits, and harms of cannabis use:

“Definitely we'd need lots for not only the staff but also for the individuals. Yeah. Because right now, I don't think there's any awareness of the different uses for cannabis. Right? Because, you know, people with pain, etc. You know, they're getting it from their friends on down the street, and it's just to get a high at this point, just to meet a need. But I think there's other needs that could really benefit” (S3202)

The above quote exemplifies staff and participant interest in trialing CSPs in MAPs in ways that promote safer use and maximize benefits, potentially beyond the impacts of reducing alcohol consumption but through other benefits such as pain management and appetite enhancement. While there was acknowledgement of the rapidly developing field of cannabis substitution and cannabis therapy evidence, clear education and training resources were requested by staff and managers as absolutely necessary to the safe implementation of CSPs in MAPs.

#### Meeting people where they are at

Organizational leaders at all sites perceived cannabis substitution as aligned with the harm reduction philosophies and principles in which MAPs operate. For example:

"I feel that the [organization] is pretty realistic with what's out there and it's person centered and, you know, we meet the people where they're at. So regardless of what substance they use, even in the [MAP] because we do have a lot of polysubstance use, we support them where they're at, in terms of being able to provide their goals or help to move them along, whichever way they want to go at that time." (S6203)

Generally, MAP staff felt that their organizational culture would support cannabis substitution. Staff often felt that cannabis substitution would naturally fall within the purview of MAPs, with many observing the current benefits of cannabis use among participants even without a formalized program. As noted in the first theme, a small minority of MAP participants reported currently using cannabis as a harm reduction tool, either through prescription or their own supply. Many staff reported that cannabis use is already occurring within programs, although without formalized program support:

"It would be a new practice I mean, you know there's… it's not [like] we haven’t tried it out in one sense. They are allowed to smoke marijuana like but they do it on the black market, right. Like they just buy it on their own and whatever. Like we're not opposed to them doing that. Right? So yeah, like they do smoke marijuana." (S2201)

At the organizational lead level, six of seven sites expressed support in principle for piloting a cannabis substitution program. Among management level staff, 60% reported clear support in principle. Sites were open to exploring CSP, although with further consultation at the organization level and with participants themselves. Most were unsure of how much this would translate into structural support at the organizational level. Despite cannabis substitution being on the radar for some programs before the feasibility study, in-depth discussions with executive leadership had not occurred in most programs due to previous challenges such as legalization and uncertainty regarding funding.

Most organizational leaders were unsure of approval requirements for implementation of a CSP. Board approval was not necessarily required for some programs, as cannabis substitution was seen as an operational decision. Some staff raised that approvals from research ethics boards, municipalities, and partnering health agencies may be necessary.

### Lack of core funding and sustainable cannabis supply (outer setting)

In regards to the outer setting of MAPs, MAP staff, managers, and organizational leaders most often noted that challenges with funding and sustainability of a cannabis supply would limit CSP implementation. According to MAP managers and organizational leaders, most MAPs currently fund alcohol through a combination of client contributions (e.g., monthly program fee), program budget, fundraising, and/or private donations. At most sites, the costs of alcohol are significantly lower than retail:

"People pay $2.50 per litre of wine whether they pay that monthly or just as they have money […] We probably subsidize a third of the costs." (S1202)

Most MAP managers and organizational leaders felt that it may be possible to re-allocate funds for alcohol to cannabis. However, a common concern voiced in all stakeholder groups was the relative cost of cannabis to alcohol:

“But again, we don't have core or sustainable funding for managed alcohol programs. Unlike all the other ones who have core and sustainable funding, we don't. So if the costs are not comparable, I can't see us purchasing that stuff [cannabis and cannabis equipment]” (S2201)

As the above quote highlights, MAPs operate with varying but often significant resource constraints. Core funding is often uncertain and multiple sources of funding are required to sustain programs. Most managers and organizational leaders felt that a pilot study would need to come with funding for the cannabis supply itself. However, six of seven organizational leaders responded that they believe their organization would be willing to submit funding applications for a cannabis supply with appropriate approval.

Beyond the question of securing funding for cannabis during the pilot study, all participant groups had concerns around funding past the duration of the study. Some MAP participants (21.1%, *n* = 4) voiced that the cost associated with procuring cannabis past the study would be difficult, particularly for legal cannabis. However, more participants (47.4%, *n* = 9) felt that after a pilot study, they would return to self-managing from their current source or independently grow their own cannabis plants.

## Discussion and recommendations

This study is one of the first we know of exploring the potential and feasibility of CSP implementation as a harm reduction intervention for people with experiences of chronic and severe AUD. CSPs have been primarily explored as an overdose prevention and opioid harm reduction strategy for people experiencing opioid dependence and structural marginalization associated with homelessness, poverty, and housing instability [51]. Reports of cannabis substitution in MAPs prior to this study have primarily included anecdotal reports, with no formalized programming.

Findings from this study support that even without formalized CSP implementation, MAP participants are engaging in cannabis substitution practices with the intent to reduce their alcohol consumption, alongside managed alcohol. These practices among MAP participants are consistent with the growing body of observational studies illustrating reductions in self-reported alcohol consumption alongside cannabis use [32–35]. Further, the strong willingness to participate in the CSP among MAP participants in this study is consistent with the recent study by Lucas et al. 2020 reporting positive associations between motivation to use cannabis for alcohol reduction and alcohol-related harms among medical cannabis users. Beyond the potential for substitution, MAP participants also anticipated that CSPs could reduce alcohol-related harms, aggression and violence, and consequences of AUD, including withdrawal, cravings, reduced appetite, and insomnia based on their personal experiences of cannabis use. While the purposive sampling method used in this study limits generalizing the prevalence of interest in CSPs across MAPs, these findings highlight their potential for people who experience severe AUD and chronic alcohol-related harms, have a history of attempting abstinence-based treatment, and often do not have access to non-stigmatizing and effective supports within abstinence-based systems [15].

MAP participants and staff identified a clear need and demand for CSPs in their respective MAPs and six of seven MAP organizational leaders endorsed support in principle for piloting a CSP. In regards to the design of the CSP intervention itself, MAP participants, managers and staff identified a partial substitution model where cannabis replaces some volume of alcohol as the most feasible model. Both MAP participants and staff spoke to the importance of choice and tailoring of CSPs to individual needs in line with harm reduction philosophy. However, existing staff support for cannabis administration would be most feasible in a self-administration model. With regard to the type of cannabis, participants and staff felt that dry cannabis via smoking would be most feasible as the currently preferred method among the majority of participants, although staff raised concerns about potential for sharing and potential issues related to the need for smoking spaces. It was apparent that participant choice to receive dry and/or oral forms of cannabis (edibles and capsules) would be important to address preferences across MAP participants and enhance uptake. While vaping was a ‘deal breaker’ for some, it is important to note that the interviews were collected mostly during a period of widespread and misleading media coverage highlighting respiratory-related deaths among young US adults vaping cannabis with Vitamin E additives [50]. In fact, when using properly regulated and safe equipment and supplies, vaping cannabis will be significantly safer than smoking and therefore should be offered as harm reduction option for future substitution programs.

In line with MAP participant and staff responses, we recommend that a pilot CSP in MAP maximize opportunities for tailored cannabis programming and additional clinical supports. A potential model could include a partial substitution of cannabis via staff administration, with participant choice to receive the cannabis or alcohol at fixed dosing times. MAP participants should also be offered to receive either dry or oral forms of cannabis according to their preference. Oral cannabis routes may come with the benefit of ease of staff administration and monitoring, however, were ‘deal breakers’ for some MAP participants. With any CSP model, clinical support will be necessary to tailor CSPs to individual preferences, goals, and clinical profiles, including identification of contraindications and monitoring of potential unintended consequences. Additional clinical and social supports are warranted for harm reduction, mental health, and other health-related counselling.

While most organizational leaders gave support in principle for a CSP, actual structural support for implementation was often unclear. MAP staff and managers stressed the need for additional funding to support human resources for the CSP, including clinical positions, staff training, and education. Managers and staff identified that funding for the cannabis supply itself would be most critical to CSP implementation and some MAP participants questioned the security of their cannabis supply past the pilot phase.

These findings highlight the precarious reality of MAPs that often operate with resource constraints and insecure, non-permanent funding. As it stands, MAPs must be creative in funding their alcohol supply, often requiring contributions from participants to support their costs. Staff and managers spoke to the higher cost of cannabis relative to alcohol as prohibitive. Challenges associated with both alcohol and cannabis supply in Canada can be partially attributed to their unique regulatory categories. Both substances are legal in Canada; however, they do not benefit from potential pharmaceutical coverage through medical regulation that could potentially address supply needs within a community MAP setting. This is despite the fact that while cannabis is legal and medically regulated, medical cannabis is not publicly funded. Thus, legalization of cannabis has reduced accessibility of cannabis to those on low incomes and highlighted an equity gap in access to and affordability of cannabis for a population who may accrue potential benefits relative to offsetting heavy drinking.

In BC, the COVID19 pandemic has prompted recommendations for managed alcohol as a safe supply intervention to reduce alcohol-related harms and promote self-isolation and physical distancing measures [52, 53]. While this places alcohol alongside other safe supply interventions for opioids (e.g., opioid-assisted therapies such as hydromorphone), stimulants, and benzodiazepines, this political recognition has not translated to secure funding for the alcohol supply itself. Unfortunately, despite the increasing recognition of both managed alcohol and cannabis substitution as harm reduction interventions, their current regulatory frameworks pose funding and supply barriers that are likely to continue to challenge CSP and MAP implementation. This will be explored elsewhere.

### Limitations

This study includes data from MAP participants, staff and managers, and organizational leaders across 6 MAPs in Canada, although with a small sample size of participants from each site. Descriptive statistics and qualitative findings were generated appropriate to feasibility objectives, although cannot be generalized for this population or across MAP sites. While this multi-site feasibility study with three distinct participant groups enhances triangulation of data from multiple sources and perspectives, the amalgamation of data may have restricted feasibility investigations specific to unique programmatic settings. Future pilot research on CSPs will be likely to include one or two sites and will explore unique programmatic contexts and factors in further depth.

## Conclusion

Cannabis substitution was considered feasible by all three participant groups and in some cases is already being used by MAP participants as a harm reduction tool. MAP participants highlighted a range of potential benefits of cannabis while also being aware of potential harms. Partial substitution of cannabis, via staff administration with participant choice to receive cannabis or alcohol at fixed times, was most preferred by all participant groups. All three groups identified a need for additional supports for implementation including peer support, staff education, and counselling. While cannabis is legal in Canada, sourcing and funding cannabis by programs were identified as primary challenges to CSP feasibility.

## Data Availability

The datasets generated and/or analyzed during the current study are not publicly available due the small sample size and associated threats to confidentiality, but are available from the corresponding author on reasonable request. Interview guides are available upon request.

## Abbreviations

AUD: Alcohol use disorder
MAP: Managed alcohol programs
CSP: Cannabis substitution program
CFIR: Consolidated framework for implementation research
CMAPS: Canadian managed alcohol program study
CBPR: Community-based participatory research
EIDGE: East side illicit drinkers group for education
VANDU: Vancouver area network of drug users
AUDIT: Alcohol use disorders inventory test
REB: Research Ethics Board
